# Nicotinic acetylcholine receptors control acetylcholine and noradrenaline release in the rodent habenulo-interpeduncular complex

**DOI:** 10.1111/bph.12841

**Published:** 2014-09-05

**Authors:** F Beiranvand, C Zlabinger, A Orr-Urtreger, R Ristl, S Huck, P Scholze

**Affiliations:** 1 Department of Pathobiology of the Nervous System, Center for Brain Research, Medical University of ViennaVienna, Austria; 2 Genetic Institute, Tel-Aviv Sourasky Medical Center and Sackler School of Medicine, Tel Aviv UniversityTel Aviv, Israel; 3 Center for Medical Statistics, Informatics, and Intelligent Systems, Medical University of ViennaVienna, Austria

## Abstract

**Background and purpose:**

Nicotinic acetylcholine receptors (nACh receptors) play a central role in the habenulo-interpeduncular system. We studied nicotine-induced release of NA and ACh in the habenula and interpeduncular nucleus (IPN).

**Experimental approach:**

The habenula and IPN were loaded with [^3^H]-choline or [^3^H]-NA and placed in superfusion chambers. [^3^H]-ACh release was also stimulated using nicotinic agonists, electrical pulses and elevated [KCl]_o_ in hippocampal and cortical slices from rats, wild-type mice and mice lacking α5, α7, β2, or β4 nACh receptor subunits. Finally, we analysed nACh receptor subtypes in the IPN using immunoprecipitation.

**Key results:**

Nicotine induced release of [^3^H]-ACh in the IPN of rats and mice. This release was calcium-dependent but not blocked by tetrodotoxin (TTX); moreover, [^3^H]-ACh release was abolished in β4-knockout mice but was unaffected in β2- and α5-knockout mice. In contrast, nicotine-induced release of [^3^H]-NA in the IPN and habenula was blocked by TTX and reduced in both β2-knockout and β4-knockout mice, and dose–response curves were right-shifted in α5-knockout mice. Although electrical stimuli triggered the release of both transmitters, [^3^H]-ACh release required more pulses delivered at a higher frequency.

**Conclusions and implications:**

Our results confirm previous findings that β4-containing nACh receptors are critical for [^3^H]-ACh release in the mouse IPN. Experiments using α5-knockout mice also revealed that unlike in the hippocampus, nicotine-induced [^3^H]-NA release in the habenulo-interpeduncular system is altered in this knockout model. As α5-containing nACh receptors play a key role in nicotine intake, our results add NA to the list of transmitters involved in this mechanism.

**Table:** Table of Links

TARGETS	LIGANDS
α2 adrenoceptor	ACh
α2 nACh receptor	Atropine
α3 nACh receptor	Bicuculline
α4 nACh receptor	Choline
α5 nACh receptor	Clorgyline
α6 nACh receptor	Cytisine
α7 nACh receptor	[3H]-epibatidine
β2 nACh receptor	Hemicholinium
β4 nACh receptor	Mecamylamine
GABAA receptor	Nicotine
Muscarinic receptor	Noradrenaline
	Tetrodotoxin
	Yohimbine

This Table lists key protein targets and ligands in this document, which are hyperlinked to corresponding entries in http://www.guidetopharmacology.org, the common portal for data from the IUPHAR/BPS Guide to PHARMACOLOGY (Pawson *et al*., 2014) and are permanently archived in the Concise Guide to PHARMACOLOGY 2013/14 (Alexander *et al*., 2013a,2013b).

## Introduction

The habenulo-interpeduncular (Hb-IPN) complex is a central relay station between forebrain and midbrain structures. The major inputs to the lateral habenula (Hb) project from the limbic system and basal ganglia motor areas, as well as brainstem areas such as the median raphe and ventral tegmental area (VTA). Ascending fibres from the lateral Hb project to the limbic forebrain, whereas descending fibres project to the median and dorsal raphe, the VTA, the substantia nigra and the locus coeruleus (LC). The medial habenula (MHb) receives descending afferents from the diagonal band of Broca and the septofimbrial and triangular nuclei; the MHb receives ascending fibres from the raphe nuclei and LC (reviewed in Klemm, 2004; Lecourtier and Kelly, 2007; Bianco and Wilson, 2009). The anatomical and functional connection between the triangular septum and the bed nucleus of the anterior commissure and the MHb was studied recently in a transgenic mouse model (Yamaguchi *et al*., 2013). Afferent fibres projecting to the MHb use the neurotransmitters GABA, glutamate, ATP and acetylcholine (ACh) (Qin and Luo, 2009), whereas efferent fibres connecting to the IPN co-release glutamate and ACh (Ren *et al*., 2011; Kobayashi *et al*., 2013).

Although the MHb is the major source of afferents projecting to the IPN, the IPN also receives descending input from the limbic forebrain and ascending input from brainstem regions such as the raphe nuclei, LC and the dorsal tegmental area (reviewed in Klemm, 2004; Bianco and Wilson, 2009; see also Kobayashi *et al*., 2013). The IPN has widespread ascending projections to limbic structures and descending projections to the dorsal tegmental area and midbrain raphe (reviewed in Klemm, 2004; Bianco and Wilson, 2009). These anatomical connections reflect the Hb-IPN's complex role as a central axial core of connectivity that links diverse forebrain and midbrain structures, suggesting that the Hb-IPN axis modulates cognition and behaviour under the control of monoaminergic centres (Klemm, 2004; Lecourtier and Kelly, 2007; Kobayashi *et al*., 2013). In humans, structural abnormalities in the Hb have been linked to schizophrenia and affective disorders, and lesion studies in rodents suggest that the habenular complex plays important roles in various behavioural domains such as cognition, anxiety, learning and memory and impulsivity (Klemm, 2004; Lecourtier and Kelly, 2007; Bianco and Wilson, 2009). More specifically, genetic deletion of the cholinergic Hb-IPN pathway showed recently that the cholinergic part of the MHb plays a crucial role in inhibitory control and cognition-dependent executive functions (Kobayashi *et al*., 2013).

Both the MHb and the IPN are unique in the CNS because of their high density of nicotinic acetylcholine receptors (nACh receptors) and because the majority of their nACh receptors contain the α3 and β4 subunits together with α2, α4, α5, α6, β2 and β3 subunits. Deleting or impairing the function of nACh receptors in the habenular complex has a large impact on nicotine dependence in rodents (and possibly humans). Hence, certain allelic variations in the gene encoding the α5 nACh receptor subunit decrease receptor function and increase susceptibility to tobacco addiction (Bierut *et al*., 2008). In rodents, α5-containing nACh receptors in the MHb may play a crucial role in controlling nicotine addiction (Salas *et al*., 2009; Fowler *et al*., 2011; Frahm *et al*., 2011). The local delivery of the putative α3β4-specific nicotinic antagonist 18-methoxycoronaridine (18-MC) into the MHb, the basolateral amygdala or the dorsolateral tegmentum decreases nicotine self-administration (Glick *et al*., 2011), whereas local delivery of 18-MC into the IPN (but not into the VTA) increases nicotine self-administration (Glick *et al*., 2011).

Here, we re-examined the subunit composition of nACh receptors in the rat and mouse IPN by immunoprecipitating [^3^H]-epibatidine-labelled receptors using custom-generated subunit-specific antibodies. We measured the receptor subunits in wild-type (WT; C57Bl/6J) mice and transgenic mice lacking the α5, β2 or β4 subunit. Therefore, our results complement and support similar studies that examined the identity of nACh receptors in the IPN of rats, WT mice, β2-knockout (KO) and β3-KO mice (Grady *et al*., 2009). We also measured the induced release of ACh from the Hb, IPN, hippocampus and cortex of both rats and mice, and of noradrenaline (NA) from the Hb, IPN and hippocampus. NA plays important roles in anxiety, stress and nicotine addiction (Bruijnzeel, 2012); thus, stressors facilitate the initiation of smoking, decrease one's motivation to quit and increase the risk of relapse after quitting. Animal studies indicate that NA – as well as other factors such as corticotropin-releasing factor, neuropeptide Y and hypocretins – plays a role in tobacco and nicotine withdrawal (reviewed in Bruijnzeel, 2012).

Our approach using both intact tissue (Hb and IPN) and slices (hippocampus and cortex) enabled us to induce transmitter release both chemically and electrically. In addition, we used mice that lack the α5, α7, β2 or β4 nACh receptor subunits. In both rats and mice, we found that activating nACh receptors in the IPN – but not in the Hb, cortex or hippocampus – drives substantial [^3^H]-ACh release. In mice, this release required the presence of β4-containing receptors, but did not require the α5, α7 or β2 subunit. In contrast, nicotine induced [^3^H]-NA release in both the Hb and IPN, and this release required the presence of receptors containing primarily β2 and β4 subunits. On the other hand, deleting the α5 subunit right-shifted the dose–response curves for nicotine-induced [^3^H]-NA release. Importantly, moderate electrical stimuli induced robust [^3^H]-NA release from both the IPN and the Hb, whereas electrically induced [^3^H]-ACh release from the IPN required a much stronger stimulation regimen. Our finding that nicotine is less potent at inducing [^3^H]-NA release in α5-KO mice adds to the complex role that α5-containing receptors play in the Hb-IPN system.

## Methods

### Animals

Transmitter-release experiments were performed using 4–8-week-old female Sprague-Dawley rats (Institute of Biomedical Research, Medical University of Vienna, Himberg, Austria). In addition, male and female 4–8-week-old WT (C57Bl/6J) mice and α5-, α7-, β2- and β4-KO mice (David *et al*., 2010) were used. In total, 1625 animals were used for these experiments (1279 mice and 346 rats). All animals were group-housed at an ambient temperature of 21°C with a light : dark regime of 10:14 h, with *ad libitum* access to standard food and water. All studies involving animals are reported in accordance with the ARRIVE guidelines for reporting experiments involving animals (Kilkenny *et al*., 2010; McGrath *et al*., 2010).

### Generation of anti-nACh receptor subunit antibodies

All antibodies were raised against the cytoplasmic loop domain of respective mouse nACh receptor subunit for the anti-α3, anti-α4, anti-α5, anti-β2 and anti-β4 antibodies (David *et al*., 2010), and for the anti-α2 and anti-α6 (Scholze *et al*., 2012) antibodies. The specificity and immunoprecipitation (IP) efficacy of these antibodies has been tested extensively (David *et al*., 2010; Scholze *et al*., 2012). We have previously reported that our anti-α6 antibody effectively precipitates striatal as well as recombinant α6β2 receptors expressed in HEK cells (Scholze *et al*., 2012). In order to exclude unspecific binding of α3-containing receptors, we now probed the antibody with superior cervical ganglion nACh receptors, known to be devoid of α6. Indeed, IP with our anti-β4 antibody precipitated 267 ± 28 fmol·mg^−1^ protein (*n* = 3) of [^3^H]-epibatidine binding sites, whereas anti-α6 was ineffective (−1.3 ± 3.4 fmol·mg^−1^; *n* = 3).

### IP of [^3^H]-epibatidine-labelled receptors

All IP experiments were performed using IPN tissue obtained from prepubescent mice and rats (17–19 days of age) following decapitation. The protocols for solubilizing, isolating and labelling the receptors with [^3^H]-epibatidine were as published previously (David *et al*., 2010; Scholze *et al*., 2012). Here, we used standardized Pansorbin cells (catalog number 507861; Calbiochem, Merck-Millipore, Darmstadt, Germany) for IP and the bicinchoninic acid protein assay reagent kit (Thermo Scientific Pierce, Rockford, IL, USA) for protein quantification.

### Superfusion experiments

Four to eight-week-old rats and mice were decapitated (the rats were anaesthetised under CO_2_) in accordance with the Guidelines of the Animal Care Committee of the Medical University of Vienna. The brains were removed immediately and placed in ice-cold low-calcium uptake buffer containing (mM): NaCl 118; KCl 4.8; CaCl_2_ 0.2; MgSO_4_ 1.2; NaHCO_3_ 25; KH_2_PO_4_ 1.2; Na_2_-EDTA 0.03; glucose 11; ascorbic acid 0.57; fumaric acid 0.5 and Na-pyruvate 5; the solution was saturated with 95% O_2_/5% CO_2_. The experiments were performed using intact tissue (Hb and IPN) or hippocampal (300 μm thickness) and parietal neocortical (400 μm thickness) slices; the slices were cut using a McIlwain tissue chopper (The Mickle Laboratory Engineering Co. Ltd, Guildford, Surrey, UK). The radiolabelled tracers were loaded by incubating the tissue for 2 h (IPN and Hb) or 1 h (slices) at 37°C in low-calcium buffer containing either 0.05 μM [^3^H]-NA (in the presence of 0.5 μM clorgyline hydrochloride and 1 mM ascorbic acid) or 0.1 μM [^3^H]-choline. After loading, the tissues were placed in a small chamber between two platinum wire electrodes and superfused for 1 h with superfusion buffer (which was identical to the low-calcium buffer, except that the [CaCl_2_] was increased to 2.5 mM). The buffer was kept at 29°C and bubbled continuously with 95% O_2_/5% CO_2_. For the [^3^H]-ACh release assays, the choline re-uptake inhibitor hemicholinium (10 μM) and atropine (0.5 μM) were added to the buffer 10 min before and throughout the sample collection. Eight samples per chamber were normally collected at 2 min intervals with a buffer flow rate of 1 mL·min^−1^. After a 6 min baseline collection period, release was triggered with electrical stimulation (100 pulses at 10 Hz, 0.5 ms, 40 V·cm^−1^, 40 mA, except where specified otherwise), a 30 s pulse of nicotine or cytisine or a 90 s pulse of elevated [KCl]. Where applicable, the antagonists mecamylamine (MCA), yohimbine and/or tetrodotoxin (TTX) were applied 10 min before and throughout the respective stimuli. To measure transmitter release in the absence of calcium, the tissues were superfused with calcium-free buffer throughout the experiment. At the end of the experiment, the radioactivity that remained in the tissue (i.e. was not released) was recovered by extraction with 1% SDS and sonication. The radioactivity in the tissue extracts and the collected perfusate samples was measured in a liquid scintillation counter.

### Calculations

**Figure 4:**
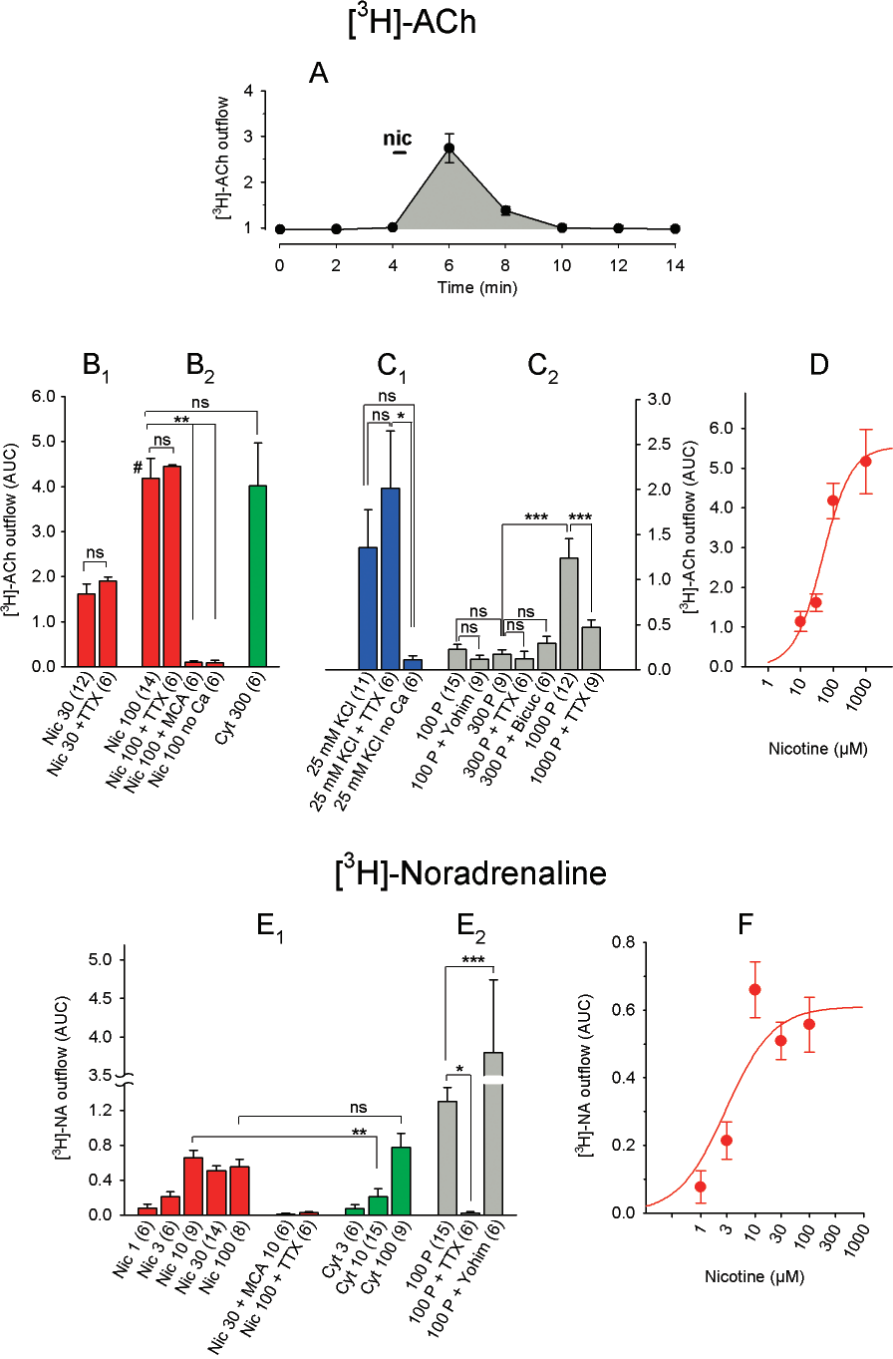
Chemically and electrically induced [^3^H]-ACh and [^3^H]-NA release from rat IPN. (A) [^3^H]-ACh release in response to a 30 s pulse of 30 μM nicotine (indicated by the horizontal bar at the 4 min time point). Atropine (0.5 μM) and hemicholinium (10 μM) were present throughout the experiment. Each data point represents the mean ± SEM of three separate IPN tissue samples. The shaded area under the curve (AUC) for this experiment was 2.2. (B) [^3^H]-ACh release in response to nicotine or cytisine. (B_1_) TTX had no significant effect on the release induced by 30 μM nicotine (*P* > 0.05, Student's *t*-test). (B_2_) Data were analysed using anova (significant with *F*_6, 49_ = 12.94 and *P* < 0.0001) and Dunnett's *post hoc* test with 100 μM nicotine as the reference data (designated by #). (C) [^3^H]-ACh release in response to high potassium or electrical pulses. (C_1_) Data were analysed using anova (significant with *F*_2, 20_ = 3.58 and *P* = 0.047) and Fisher's least significant difference (LSD) test for pairwise group comparisons. (C_2_) Data were analysed by one-way anova (significant with *F*_6, 59_ = 12.60 and *P* < 0.0001), followed by Tukey's *post hoc* test for pairs of datasets. (D) Dose–response curve of nicotine-induced [^3^H]-ACh release. Nicotine EC_50_: 47.9 μM, maximum release effect (AUC): 5.5. Each data point represents ≥9 separate measurements. (E) [^3^H]-NA release in response to the indicated stimuli. (E_1_)Student's unpaired *t*-test was applied for a comparison of data with equal agonist concentrations. (E_2_) Release data were analysed by one-way anova (significant with *F*_2, 24_ = 16.70 and *P* < 0.0001) and pairwise group comparisons using Fisher's LSD test. (F) Dose–response curve of nicotine-induced [^3^H]-NA release. Nicotine EC_50_: 2.9 μM; maximum effect (AUC): 0.61. Each data point represents ≥6 separate measurements. All data are presented as the mean ± SEM. All concentrations are in μM, and the numbers in parentheses indicate the number of measurements. Bicuc, 30 μM bicuculline; Cyt, cytisine; MCA, 10 μM mecamylamine; Nic, nicotine; TTX, 1 μM tetrodotoxin; Yohim, 2 μM yohimbine; 100 P, 100 pulses (0.5 ms, 10 Hz, 40 mA). ns, *P* > 0.05; ***P < 0.001; **P < 0.01; *P < 0.05.

The radioactive content (measured as cpm) of a given fraction was divided by the total radioactivity at the start of the corresponding 2 min collection period. Accordingly, the radioactivity of all subsequent fractions was added to the radioactivity retained in the tissue. Baseline release was calculated as the average of three fractions before a stimulus. Stimulation-evoked release was calculated by normalizing to baseline (i.e. release during and after a stimulus divided by baseline, assuming that basal release is linear). We quantified stimulation-evoked release using the AUC feature in GraphPad Prism (version 5.00 for Windows, GraphPad Software, San Diego, CA, USA, http://www.graphpad.com; see Figure 4A).

All summary data are presented as mean ± SEM. Group differences were analysed using Student's unpaired *t*-test (for a comparison between two related datasets) or one-way anova to test the global hypothesis of no differences in true mean values between groups. If this test was significant at α = 0.05, four (or more) datasets were analysed using *post hoc* multiple comparison tests (Tukey's for pairs of datasets or Dunnett's for comparing datasets with a reference group). Datasets consisting of three groups were compared pair-wise using Fisher's least significant difference test: when comparing only three groups, no further adjustment for multiple testing is required when the global null hypothesis was rejected. Dose–response curves for the agonists were fitted using non-weighted non-linear regression to the dose–response curve (GraphPad Prism). An *F*-test was used to test for different versus shared (identical) EC_50_ parameter values. Differences are considered significant if *P* < 0.05.

### Materials

The following materials and reagents were obtained from the following sources: levo-[ring-2,5,6-[^3^H]-noradrenaline ([^3^H]-NA, 250 Ci mmol^−1^): PerkinElmer (Waltham, MA, USA); [methyl-^3^H]-choline chloride, 60 Ci mmol^−1^: ARC American Radiolabeled Chemicals, Inc (St. Louis, MO, USA); TTX HCl: Latoxan (Valence, France); atropine (A0257), hemicholinium (H108), (-)-nicotine (N3876), MCA hydrochloride (M9020), ascorbic acid (A4544), yohimbine (Y3125) and clorgyline hydrochloride (M3778): Sigma-Aldrich (St. Louis, MO, USA). All other chemicals were obtained as analytical grade from Merck (Darmstadt, Germany). The drug/molecular target nomenclature conforms to BJPs Concise Guide to Pharmacology (Alexander *et al*., 2013).

## Results

### Subunit composition of nACh receptors in the rat IPN

**Figure 1:**
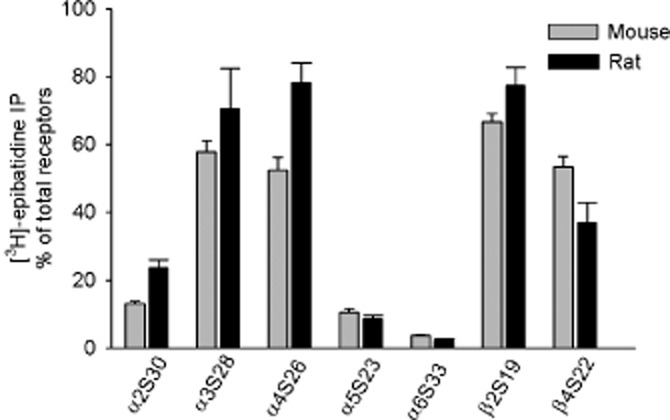
Subunit composition of nACh receptors in the mouse and rat interpeduncular nucleus (IPN). The IPN of P18 wild-type (C57Bl/6J) mice or wild-type (Sprague-Dawley) rats were solubilized, and the nACh receptors were labelled with 1 nM [^3^H]-epibatidine, then immunoprecipitated with the subunit-specific antibodies indicated. Non-specific binding was measured in the presence of 300 μM nicotine and subtracted from the total in order to obtain the specific binding depicted in the graph. A value of 100% is based on the combined use of anti-β2 and anti-β4 antibodies, which precipitates all hetero-oligomeric receptors. The data are represented as the mean ± SEM of three (rat) and four (mouse) independent experiments, each of which was performed in duplicate.

**Figure 2:**
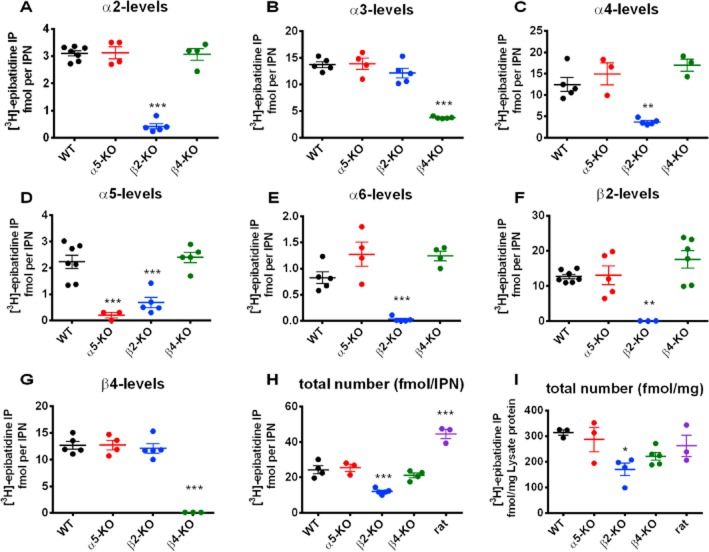
Quantification of distinct nACh receptors in the IPN of wild-type mice (WT), α5-, β2-, and β4-KO mice and wild-type rats. The IPN from the indicated animals were solubilized and the nACh receptors were labelled with 1 nM [^3^H]-epibatidine and immunoprecipitated with the indicated subunit-specific antibody. Non-specific binding was measured in the presence of 300 μM nicotine and subtracted from each total in order to obtain the specific binding shown in the plots. Each data point represents an experiment performed in duplicate. Horizontal and vertical lines show the mean ± SEM, respectively, of three to five independent experiments. Data were analysed using the one-way anova followed by Dunnett's *post hoc* tests (values compared with wild-type mice). Headings in each panel designate nACh receptors precipitated using the corresponding subunit-specific antibodies. The total number of receptors (panels H and I) was assessed by the combined use of anti-β2 and anti-β4 antibodies. WT: C57BL/6J mice; α5-KO, β2-KO and β4-KO: α5-knockout, β2-knockout and β4-knockout mice respectively. Levels of significance for global anova were: *F*_3, 16_ = 83.13, *P* < 0.0001 (for α2); *F*_3, 15_ = 46.70, *P* < 0.0001 (for α3); *F*_3, 12_ = 16.36, *P* = 0.0002 (for α4); *F*_3, 16_ = 19.44, *P* < 0.0001 (for α5); *F*_3, 14_ = 22.10, *P* < 0.0001 (for α6); *F*_3, 17_ = 9.96, *P* = 0.0005 (for β2); *F*_3, 13_ = 47.17, *P* < 0.0001 (for β4); *F*_4, 14_ = 44.84, *P* < 0.0001 (for fmol per IPN); *F*_4, 13_ = 4.08, *P* = 0.0233 (for fmol·mg^−1^ protein). Panels show only significant differences: ***P < 0.001, **P < 0.01, *P < 0.05.

We labelled solubilized nACh receptors with [^3^H]-epibatidine, then performed IP using subunit-specific antibodies. We found that the rat IPN contains high levels of receptors containing the α2, α3, α4, β2 and β4 subunits and lower (but detectable) levels of α5 and α6 subunits (Figure 1). The total number of receptors was 262.2 ± 41.3 fmol·mg^−1^ protein (44.5 ± 2.6 fmol per IPN; *n* = 3; Figure 2H,I). In addition, the rat IPN contained approximately twice as many β2-containing receptors as β4-containing receptors (Figure 1).

### Subunit composition of nACh receptors in the mouse IPN

The total number of receptors was 24.3 ± 2.2 fmol per IPN (313.6 ± 10.4 fmol mg^−1^ protein; *n* = 4; Figure 2H,I; which is on par with the rat IPN). The majority of α3 subunits were co-assembled with β4, as the level of α3 subunits was significantly lower in the β4-KO mice (3.7 ± 0.1 fmol per IPN; *n* = 4) compared with WT mice (13.7 ± 0.5 fmol per IPN; *n* = 5) (Figure 2). The number of α2-, α4-, α5- and α6-containing receptors was similar between WT and β4-KO mice. The number of β2-containing receptors was higher – albeit not statistically significant – in the β4-KO mice (17.6 ± 2.5; *n* = 6) compared with WT mice (12.7 ± 0.6; *n* = 7) (Figure 2).

**Figure 3:**
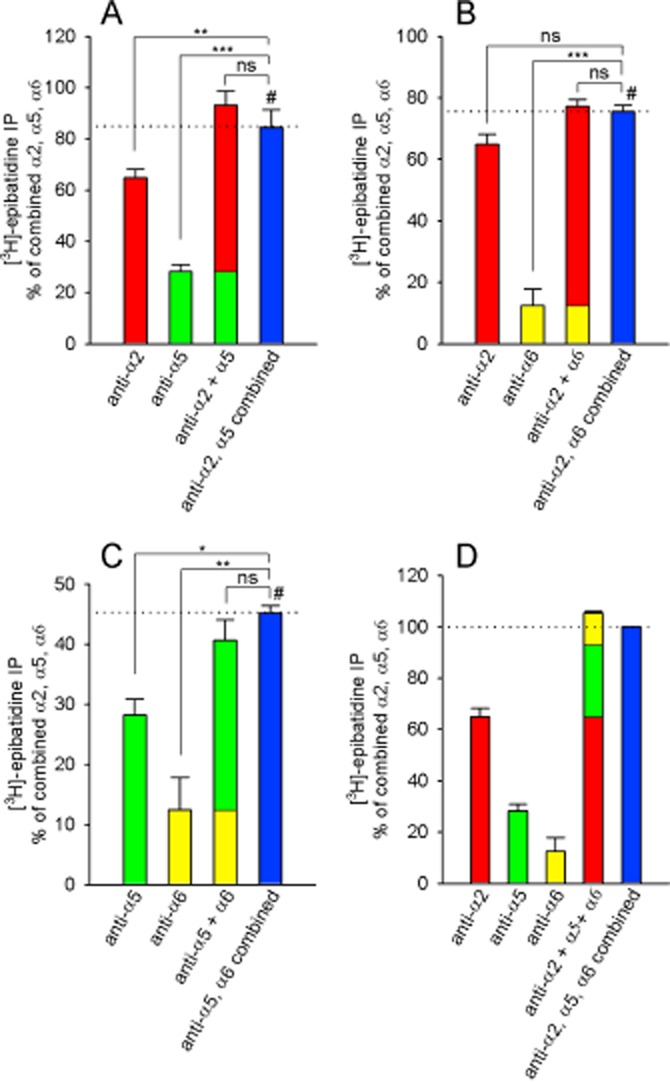
α2 and α5, and α5 and α6 nACh receptor subunits do not co-assemble. [^3^H]-epibatidine-labelled nACh receptors were immunoprecipitated using anti-α2, anti-α5 or anti-α6 antibodies. In three identically designed assays, the receptors were precipitated by each antibody alone and by combining either two (panels A–C) or all three antibodies (panel D). Each bar represents the mean percentage of [^3^H]-epibatidine-labelled receptors that were precipitated by the antibodies in relation to the combined use of all three antibodies (100% in panel D). Where indicated, the number of receptors immunoprecipitated by each individual antibody differed significantly from the number of receptors precipitated by a combination of the antibodies (the blue bars in all panels). The data are presented as the mean percentage ± SEM (relative to the combined precipitation using the anti α2, α5 and α6 antibodies, see panel D) of three independent experiments performed in duplicate. The data were analysed using repeated one-way anova followed by a Dunnett's multiple comparison *post hoc* test with data referenced to the result obtained by immunoprecipitation with combined antibodies, indicated by #). Levels of significance for global anova were: *F*_3, 6_ = 161.2 and *P* < 0.0001 (panel A), *F*_3, 6_ = 58.3 and *P* < 0.0001 (panel B), *F*_3, 6_ = 18.6 and *P* = 0.0019 (panel C). ns, *P* > 0.05, *P < 0.05, **P < 0.01, ***P < 0.001.

The β2-KO mice contained significantly fewer nACh receptors compared with WT mice (Figure 2H,I); specifically, the number of α2-, α4-, α5- and α6-containing receptors was lower in the β2-KO mice; however, the number of α3- and β4-containing receptors was similar to WT (Figure 2). Because the number of α2, α5 and α6 subunits was reduced in the β2-KO IPN, we examined whether these subunits co-assemble into one receptor (i.e. receptors containing α2 and α5, α5 and α6, or α2 and α6). We, therefore, immunoprecipitated receptors from WT mice using the anti-α2, anti-α5 or anti-α6 antibody alone or in combination (Figure 3). We then compared the results obtained using individual antibodies with the results obtained using a mixture of the same antibodies and found significant differences for anti-α5 combined with either anti-α2 or anti-α6 (Figure 3). Furthermore, when comparing the sum of anti-α5 and anti-α2 (Figure 3A) and the sum of anti-α5 and anti-α6 (Figure 3C) with the results obtained using a mixture of the same two antibodies, we found no difference. These data imply that α5 does not co-assemble with either α2 or α6 (Figure 3).

### [^3^H]-ACh and [^3^H]-NA release from the rat IPN

We next examined the release of ACh ([^3^H]-ACh) and NA ([^3^H]-NA) from rat IPN upon stimulation of nACh receptors (Figure 4). Nicotine induced [^3^H]-ACh release with an EC_50_ of 47.9 μM and a maximum release (measured using the AUC method) of 5.5 (Figure 4B,D). The effect of cytisine, a full agonist of β4-containing receptors, was similar to nicotine (Figure 4B), suggesting that β4-containing receptors mediate the majority of nicotine-induced release. Nicotine-induced [^3^H]-ACh release required extracellular calcium and was blocked completely in the presence of the nACh receptor antagonist MCA (Figure 4B). On the other hand, [^3^H]-ACh release was not dependent on action potentials, as it was not blocked by the sodium channel blocker TTX (Figure 4B).

Unlike nicotine-induced release, the release of [3H]-ACh using standard electrical field pulses (100–300 pulses at 10 Hz) was relatively weak (AUC: 0.23), and this release was not significantly reduced in the presence of TTX (Figure 4C). Because applying sustained tetanic stimulation (e.g. 20 s at 50 Hz) to cholinergic IPN afferents produces nACh receptor-mediated slow inward currents in IPN neurons (Ren *et al*., 2011), we increased the number and frequency of pulses to 1000 pulses at 50 Hz. This stimulation protocol increased the maximum release of [^3^H]-ACh to 1.2 ± 0.2, and this release was partially blocked by TTX (Figure 4C). Increasing extracellular [KCl] from 4.8 to 25 mM moderately increased [^3^H]-ACh release (AUC: 1.4), and this release required extracellular calcium but was not blocked by TTX (Figure 4C).

We found strikingly different effects with respect to nicotine-induced [^3^H]-NA release from the IPN. Although the effects of nicotine on [^3^H]-NA release were much smaller than on [^3^H]-ACh (AUC: 0.56 ± 0.08 with 100 μM nicotine), the release of [^3^H]-NA was blocked completely by TTX (Figure 4E). Cytisine, a partial agonist of β2-containing receptors, had the same effect as nicotine at 100 μM, although 10 μM cytisine induced significantly less release than 10 μM nicotine (*P* < 0.01, Student's unpaired *t*-test; Figure 4E). The EC_50_ and maximum nicotine-induced [^3^H]-NA release were 2.9 μM and 0.61 respectively (Figure 4F). In contrast with [^3^H]-ACh release, electrically induced [^3^H]-NA release was considerably greater than nicotine-induced [^3^H]-NA release (*P* < 0.01, Student's unpaired *t*-test; comparison between release induced by 100 pulses and release induced by 10 μM nicotine) and was blocked completely by TTX (Figure 4E).

**Figure 6:**
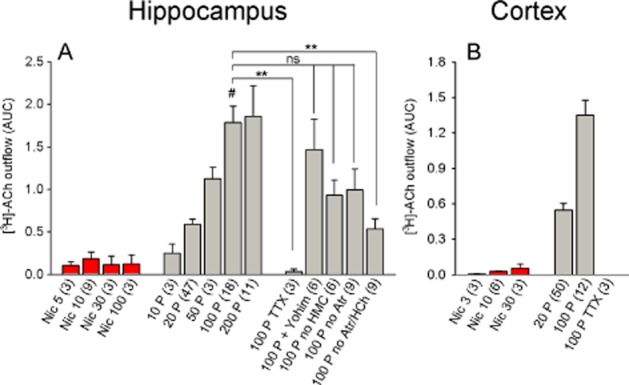
Nicotine-induced and electrically induced [^3^H]-ACh release from rat hippocampal and parietal cortical slices. (A and B) [^3^H]-ACh release from hippocampal slices (A) and cortical slices (B) in response to the indicated stimuli. Nicotine-induced release was small in both the hippocampus and the cortex and was therefore not analysed further. The data for electrically induced [^3^H]-ACh release in panel (A) were analysed by one-way anova (significant with *F*_5, 57_ = 5.07 and *P* = 0.0007), followed by Dunnett's *post hoc* test (referenced to 100 pulses; indicated by #). (B) TTX fully prevented electrically induced release in cortical slices. The data are presented as mean ± SEM. All nicotine concentrations are in μM; the numbers in parentheses represent the number of measurements. Atr, atropine; HMC, 10 μM hemicholinium; Nic, nicotine; TTX: 1 μM tetrodotoxin; Yohim, 2 μM yohimbine; 100 P, 100 pulses (0.5 ms, 10 Hz, 40 mA). ns, *P* > 0.05; **P < 0.01.

Given that electrical stimuli evoked relatively weak [^3^H]-ACh release, we tested whether simultaneously releasing endogenous NA or GABA might inhibit this ACh release. Neither the GABA_A_ receptor antagonist bicuculline nor the α_2_ adrenoceptor antagonist yohimbine increased the release of [^3^H]-ACh in the IPN (Figure 4C). Similarly, yohimbine had no effect on electrically induced [^3^H]-ACh release in the hippocampus (see Figure 6A). In contrast, yohimbine increased electrically induced [^3^H]-NA release in the IPN (Figure 4E).

### [^3^H]-ACh and [^3^H]-NA release from the rat Hb

**Figure 5:**
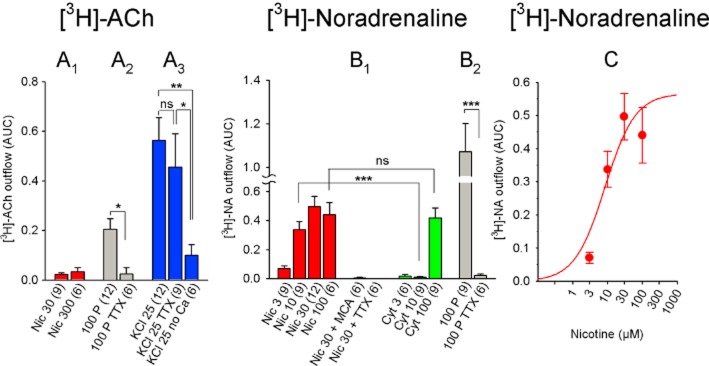
Chemically and electrically induced [^3^H]-ACh and [^3^H]-NA release from rat habenula. (A) [^3^H]-ACh release in response to the indicated stimuli. (A_1_) Due to low levels, release in response to nicotine was not investigated further for TTX sensitivity or dependence on calcium. (A_2_) Difference was analysed by Student's unpaired *t*-test. (A_3_) Release data were analysed by one-way anova (significant with *F*_2, 24_ = 4.25 and *P* = 0.026) and Fisher's least significant difference test for pairwise group comparisons. (B) [^3^H]-NA release in response to the indicated stimuli. (B_1_) Student's unpaired *t*-test was applied for a comparison of data with equal agonist concentrations. Note that 10 μM nicotine was less efficacious than 10 μM cytisine, whereas the effect of 100 μM did not differ significantly from 100 μM cytisine. (B_2_) Difference was analysed by Student's unpaired *t*-test. (C) Dose–response curve of nicotine-induced [^3^H]-NA release. Nicotine EC_50_: 8.3 μM, maximum release (AUC): 0.57. Each data point represents ≥6 separate measurements. All data are presented as mean ± SEM. All concentrations are in μM; the numbers in parentheses indicate the number of measurements. Cyt, cytisine; Nic, nicotine; MCA, 10 μM mecamylamine; TTX, 1 μM tetrodotoxin; 100 P, 100 pulses (0.5 ms, 10 Hz, 40 mA); 25 KCl: 25 mM KCl. ns, *P* > 0.05; ***P < 0.001; **P < 0.01; *P < 0.05.

We next examined the release of [^3^H]-ACh and [^3^H]-NA from the rat Hb (Figure 5). In contrast with the IPN, nicotine induced virtually no [^3^H]-ACh release in the Hb (AUC: 0.03; Figure 5A). As in the IPN, electrically induced [^3^H]-ACh release was low in the Hb (AUC: 0.2), but was TTX-sensitive (Figure 5A). On the other hand, high-KCl-induced [^3^H]-ACh release was smaller in the Hb (AUC: 0.56) than in the IPN, and was calcium-dependent but TTX-insensitive (Figure 5A).

The EC_50_ and maximum nicotine-induced [^3^H]-NA release from the Hb were 8.3 μM and 0.57, respectively, which are similar to the IPN (Figure 5B,C). The nicotine-induced release of [^3^H]-NA in the Hb was blocked completely by MCA and TTX (Figure 5B). Similar to the IPN, electrically induced [^3^H]-NA release was significantly larger than nicotine-induced release (*P* < 0.001, Student's unpaired *t*-test; comparison between release induced by 100 pulses and release induced by 30 μM nicotine); moreover, cytisine- and nicotine-induced release were similar at 100 μM, whereas cytisine induced significantly less release than nicotine at 10 μM (*P* < 0.001, Student's unpaired *t*-test; Figure 5B).

### Release of [^3^H]-ACh in the rat hippocampus and cortex

We next measured the release of [^3^H]-ACh in the parietal cortex and hippocampus in order to compare its release with the IPN and Hb. Nicotine induced extremely little [^3^H]-ACh release from hippocampal slices (AUC: 0.18; Figure 6A) and cortical slices (AUC: 0.05; Figure 6B). In contrast, our standard electrical stimulation protocol (i.e. 100 pulses) induced robust [^3^H]-ACh release from both the hippocampus and cortex (AUC: 1.8 and 1.4, respectively; Figure 6A, B), a response that was nearly 10-fold larger than the Hb and IPN. The release of [^3^H]-ACh increased approximately linearly with increasing pulse number, and the release induced with 100 pulses was blocked completely by TTX in both the hippocampus (Figure 6A) and cortex (Figure 6B). The α_2_ adrenoceptor antagonist yohimbine had no effect on electrically induced [^3^H]-ACh release in the hippocampus (Figure 6A). Moreover, omitting both the muscarinic receptor antagonist atropine and the choline re-uptake inhibitor hemicholinium from the bath decreased electrically induced [^3^H]-ACh release by approximately 50% (*P* < 0.01; one-way anova with Dunnett's *post hoc* test; Figure 6A).

### [^3^H]-ACh and [^3^H]-NA release in the mouse IPN

**Figure 7:**
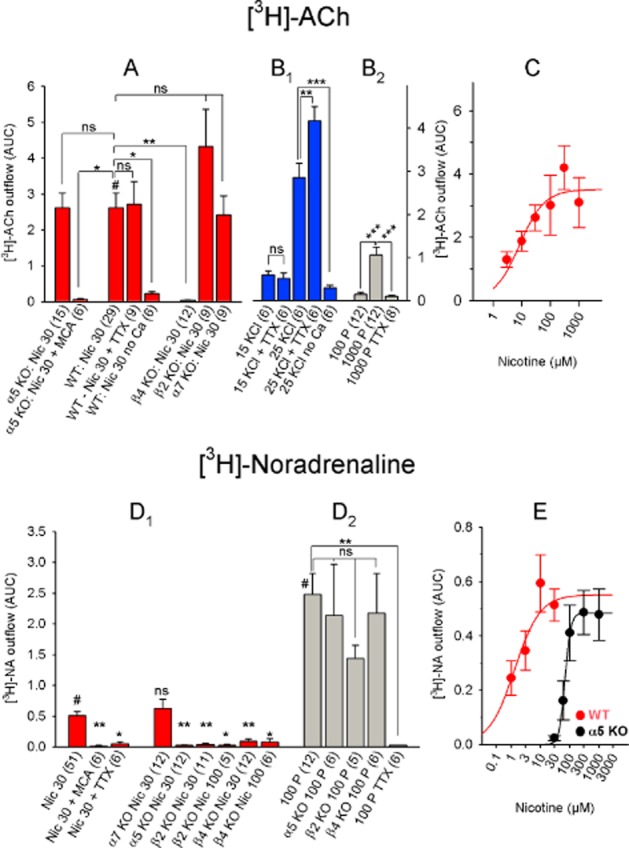
Chemically and electrically induced [^3^H]-ACh and [^3^H]-NA release in mouse IPN. (A) [^3^H]-ACh release in response to nicotine. Differences were analysed by one-way anova (significant with *F*_7, 87_ = 6.44 and *P* < 0.0001), followed by Dunnett's *post hoc* test (referenced to WT 30 μM nicotine; indicated by #). (B) [^3^H]-ACh release in response to the indicated stimuli. (B_1_) Differences were analysed by one-way anova (significant with an *F*_4, 25_ = 62.37 and a corresponding *P* value < 0.0001), followed by Tukey's *post hoc* test for pairs of datasets. (B_2_) Differences were analysed by one-way anova (significant with *F*_2, 29_ = 18.36 and *P* < 0.0001) and Fisher's least significant difference test for pairwise group comparisons. (C) Dose–response curve of nicotine-induced [^3^H]-ACh release. Nicotine EC_50_: 8.3 μM, maximum effect (AUC): 3.5. Each data point represents ≥6 separate measurements. (D) [^3^H]-NA release in response to the indicated stimuli. (D_1_) Data were analysed by one-way anova (significant with *F*_8, 112_ = 8.42 and *P* < 0.0001), followed by Dunnett's *post hoc* test (referenced to WT 30 μM nicotine; indicated by #). (D_2_) Data in response to electrical stimuli were analysed using one-way anova (significant with *F*_4, 30_ = 4.01 and *P* = 0.010), followed by Dunnett's *post hoc* test (referenced to 100 pulses; indicated by #). (E) Dose–response curve of nicotine-induced [^3^H]-NA release. Data from WT mice are shown: nicotine EC_50_: 1.34 μM (confidence interval 0.38–4.71 μM); maximum effect: 0.54; Hill coefficient 1.3. With data from α5-knockout mice: nicotine EC_50_: 69.24 μM (confidence interval 52.25–91.76 μM); maximum effect: 0.48; Hill coefficient: 4.4. Although conspicuous, the shift in the dose–response curve is not significant (*F*_1,113_ = 1.218 and *P* = 0.272). Each data point represents ≥6 separate measurements. The data are presented as mean ± SEM. All concentrations are in μM; the numbers in parentheses represent the number of measurements. MCA, 10 μM mecamylamine; Nic, nicotine; TTX, 1 μM tetrodotoxin; 100 P, 100 pulses (0.5 ms, 10 Hz, 40 mA); 15 KCl and 25 KCl: 15 mM and 25 mM KCl respectively. WT: C57BL/6J mice; α5 KO, α7 KO, β2 KO and β4 KO: α5-knockout, α7-knockout, β2-knockout and β4-knockout mice respectively. ns, *P* > 0.05; ***P < 0.001; **P < 0.01; *P < 0.05.

**Figure 9:**
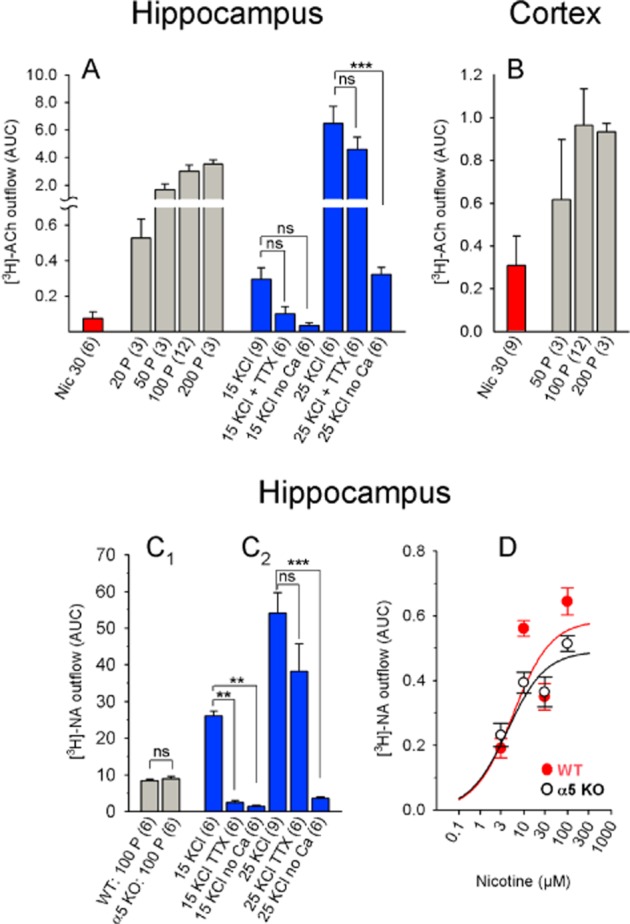
Chemically and electrically induced [^3^H]-ACh and [^3^H]-NA release from mouse hippocampal and parietal cortical slices. (A and B) [^3^H]-ACh release from hippocampal (A) and cortical slices (B) in response to the indicated stimuli. Differences in KCl-induced release (panel A) were analysed using one-way anova (significant with *F*_5, 33_ = 22.75 and *P* < 0.0001), followed by Tukey's *post hoc* test for pairs of datasets. (C) [^3^H]-NA release from hippocampal slices in response to the indicated stimuli. (C_1_) Data were analysed using Student's *t*-test (100 pulses; WT vs. α5 knockout). (C_2_) Data were analysed using one-way anova (significant with *F*_5, 33_ = 29.85 and *P* < 0.0001), followed by Tukey's *post hoc* test. The graph shows statistical comparisons of interest. (D) Dose–response curve of nicotine-induced [^3^H]-NA release. With data from WT mice: nicotine EC_50_: 5.60 μM (confidence interval 0.94–33.14 μM); maximum effect: 0.58; Hill coefficient: 1.0). With data from α5-knockout mice: nicotine EC_50_: 4.21 μM (confidence interval 1.07–16.59); maximum effect: 0.49; Hill coefficient: 1.0. EC_50_ values do not differ significantly (*F*_2, 55_ = 0.42 and *P* = 0.66). The data are presented as mean ± SEM. All nicotine concentrations are in μM; the numbers in parentheses represent the number of measurements. Nic, nicotine; TTX, 1 μM tetrodotoxin; 100 P, 100 pulses (0.5 ms, 10 Hz, 40 mA); 15 KCl, 25 KCl: 15 mM KCl and 25 mM KCl respectively. WT: C57BL/6J mice; α5 KO: α5-knockout mice. ns, *P* > 0.05; ***P < 0.001; **P < 0.01.

We next examined [^3^H]-ACh and [^3^H]-NA release in the mouse IPN and Hb. In the IPN, nicotine induced [^3^H]-ACh release with an EC_50_ and maximum release of 8.3 μM and 3.5 respectively (Figure 7C). Thus, nicotine was more potent – but less efficacious – at driving ACh release in the mouse IPN compared with the rat IPN (hypothesis of shared EC_50_ rejected with *P* < 0.05; hypothesis of shared maximum rejected with *P* < 0.01; *F*-test). However, the general properties of [^3^H]-ACh release were similar to the rat. Specifically, (i) nicotine-induced [^3^H]-ACh release was TTX-insensitive but required extracellular calcium (Figure 7A); (ii) the response to our standard pulse protocol was small (AUC: 0.15 with 100 pulses; 1.3 with 1000 pulses; Figure 7B); and (iii) increasing extracellular KCl from 4.8 to 25 mM increased [^3^H]-ACh release in a partially calcium-dependent, TTX-insensitive manner (Figure 7B). In addition, increasing extracellular KCl from 4.8 to 15 mM induced only marginal [^3^H]-ACh release in the IPN; however, unlike the hippocampus (Figure 9A), this release was not TTX-sensitive (Figure 7B).

The availability of KO mice with deletions of specific nACh receptor subunits enabled us to identify which receptor subtypes mediate [^3^H]-ACh release. [^3^H]-ACh release in the IPN of α5-, α7- and β2-KO mice was similar to WT levels; however, release was abolished in the IPN of β4-KO mice (Figure 7A).

We observed several similarities regarding [^3^H]-NA release between the mouse and rat IPN. Specifically, the EC50 and maximum release induced by nicotine in the mouse IPN were 1.34 μM and 0.54, respectively (Figure 7E), and nicotine-induced release was blocked by both MCA and TTX. Similarly, electrically induced [^3^H]-NA release was significantly larger (AUC: 2.5) than nicotine-induced release (Figure 7D). Interestingly, the β2- and β4-KO mice had nearly undetectable nicotine-induced [^3^H]-NA release (Figure 7D), suggesting that each of the two subunits is necessary – but not sufficient – for mediating nicotine-induced [^3^H]-NA release in the mouse IPN. In contrast, deleting the α5 subunit increased the Hill coefficient (from 1.3 to 4.4) and rendered nACh receptors less sensitive to nicotine, causing a 50-fold increase in EC_50_ (Figure 7E). On the other hand, α7 subunits do not appear to play a role in nicotine-induced [^3^H]-NA release (Figure 7D). The lack of release in the β2- and β4-KO animals can be attributed specifically to altered nACh receptors, not to the transmitter release machinery, as all three KO mice (α5-, β2- and β4-KO) had WT levels of electrically induced [^3^H]-NA release (Figure 7D).

### [^3^H]-ACh and [^3^H]-NA release in the mouse Hb

**Figure 8:**
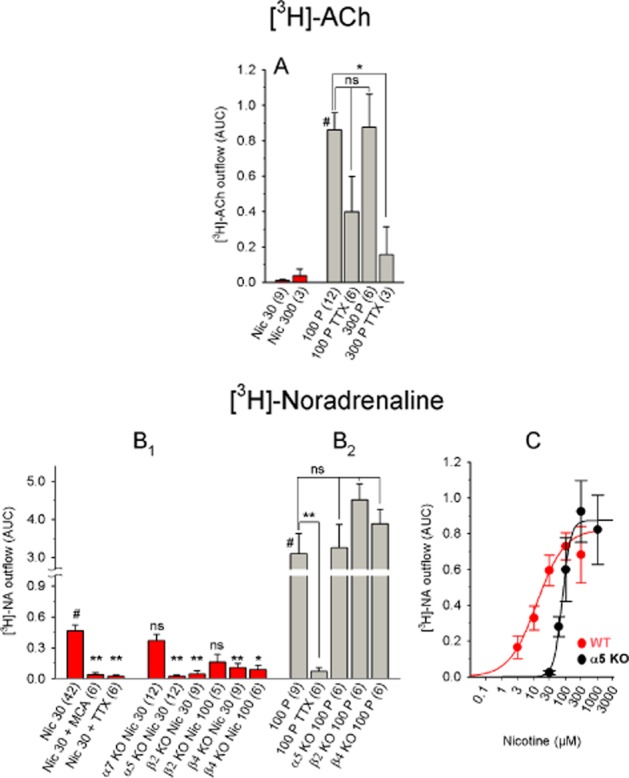
Chemically and electrically induced [^3^H]-ACh and [^3^H]-NA release from the mouse habenula. (A) [^3^H]-ACh release in response to the indicated stimuli. Nicotine-induced release was small and therefore not analysed further. The release data in response to electrical stimuli were analysed using one-way anova (significant with *F*_3, 23_ = 4.05 and *P* = 0.019), followed by Dunnett's *post hoc* test (referenced to 100 pulses; indicated by #). (B) [^3^H]-NA release in response to the indicated stimuli. (B_1_) Differences were analysed using one-way ANOVA (significant with an *F*_8, 98_ = 6.16 and *P* < 0.0001), followed by Dunnett's *post hoc* test (referenced to WT 30 μM nicotine; indicated by #). (B_2_) Differences were analysed using one-way anova (significant with *F*_4, 28_ = 12.11 and *P* < 0.0001), followed by Dunnett's *post hoc* test (referenced to WT 100 pulses; indicated by #). (C) Dose–response curve of nicotine-induced [^3^H]-NA release. With data from WT mice: nicotine EC_50_: 11.87 μM (confidence interval: 2.93–48.07 μM); maximum effect: 0.80; Hill coefficient: 1.1). With data from α5-knockout mice. nicotine EC_50_: 77.3 μM (confidence interval: 55.2–108.4 μM); maximum effect: 0.87; Hill coefficient: 3.3). The shift in the dose–response curve is not significant (*F*_1, 112_ = 0.79 and *P* = 0.375). Each data point represents ≥6 separate measurements. The data are presented as mean ± SEM. All concentrations are in μM; the numbers in parentheses represent the number of measurements. MCA, 10 μM mecamylamine; Nic, nicotine; TTX, 1 μM tetrodotoxin; 100 P, 100 pulses (0.5 ms, 10 Hz, 40 mA). WT: C57BL/6J mice; α5 KO, α7 KO, β2 KO and β4 KO: α5-knockout, α7-knockout, β2-knockout and β4-knockout mice respectively. ns, *P* > 0.05; **P < 0.01; *P < 0.05.

Similar to the rat, stimulating the mouse Hb with 100 electrical pulses (AUC: 0.86) was more effective at releasing [^3^H]-ACh than stimulating with 300 μM nicotine (AUC: 0.04) (Figure 8A). Due to nicotine's extremely low efficacy, we did not test MCA, TTX or calcium-free buffer; however, electrically induced [^3^H]-ACh release was inhibited by TTX (Figure 8A).

Nicotine-induced release of [^3^H]-NA in the Hb was qualitatively similar to nicotine-induced [^3^H]-NA release in the IPN. Specifically, the EC50 and maximum release were 11.87 μM and 0.8, respectively (Figure 8C), and release was blocked by both MCA and TTX (Figure 8B). Likewise, β2- and β4-KO mice had very little nicotine-induce [^3^H]-NA release, whereas the release in α7-KO mice was similar to WT (Figure 8B). Deleting the α5 subunit increased the Hill coefficient (from 1.1 to 3.3) and shifted the EC_50_ of nicotine-induced release (to 77.3 μM, Figure 8C). Compared with nicotine-induced release, the [^3^H]-NA release in response to electrical pulses was quite large (AUC: 3.1 with 100 pulses in WT mice) and not affected in any of the KO mice (Figure 8B).

### [^3^H]-ACh and [^3^H]-NA release in the mouse hippocampus and cortex

In contrast to the rat experiments, in mouse slices, 30 μM nicotine was more effective at inducing [^3^H]-ACh release from the cortex (AUC: 0.3) than from the hippocampus (AUC: 0.07; Figure 9A,B). On the other hand, 100 electrical pulses triggered more [^3^H]-ACh release in the hippocampus (AUC: 3.0) than in the cortex (AUC: 0.96; Figure 9A,B). Increasing extracellular [KCl] to 15 mM increased [^3^H]-ACh in a partial TTX-sensitive manner, whereas 25 mM KCl caused considerably higher release that was TTX-insensitive (Figure 9A).

Given that deleting the α5 subunit affected nicotine-induced [^3^H]-NA release from mouse IPN and Hb, we also studied [^3^H]-NA release in the mouse hippocampus. Interestingly, we found no difference between WT and α5-KO mice (Figure 9D). In addition, [^3^H]-NA release was extremely robust in response to both electrical stimuli and elevated [KCl] (Figure 9C). As with [^3^H]-ACh, 15 mM KCl-induced [^3^H]-NA release – but not 25 mM KCl-induced release – was TTX-sensitive and both conditions were highly calcium-dependent (Figure 9C).

## Discussion and conclusions

### nACh receptor receptor subtypes in the rat and mouse IPN

The rich cholinergic innervation of the IPN is well documented (reviewed in Klemm, 2004). In the IPN, nACh receptors are located at both presynaptic and postsynaptic sites (Mulle *et al*., 1991; Grady *et al*., 2001,2009). In contrast with a previous IP study in which β4-containing receptors outnumbered β2-containing receptors in the rat (Grady *et al*., 2009), we found more β2-containing receptors than β4-containing receptors (at a ratio or approximately 2:1), and we found that approximately 20% of nACh receptors in the rat IPN contain both β2 and β4 subunits. We also detected high levels of α2 subunits (particularly in the rat) and relatively low levels of α5 and α6 subunits. We did not measure β3 subunits, which can assemble with β4 and affect ACh release in the IPN (Grady *et al*., 2009). In partial agreement with Grady *et al*. (2009), the β2-KO mice had no detectable α6 subunits, significantly reduced α2, α4 and α5 subunits, and WT levels of α3 and β4 subunits. In mice, deleting the β4 subunit significantly reduced – but did not abolish – the number of α3-containing receptors, indicating that approximately 73% of the α3 subunits assemble into β4-containing receptors and approximately 27% assemble into β2-containing receptors. We found no change in the levels of α2, α4, α5 or α6 subunits in the β4-KO mice. From our experiments with β2-KO and β4-KO mice, we conclude that α5 subunits co-assemble with β2 – but not β4 – subunits in the mouse IPN, as previously suggested (Grady *et al*., 2009). Our IP assays based on the combined use of anti-α2 and anti-α5 antibodies (Figure 3) rule out the possibility that these two subunits co-assemble in one receptor. However, we currently have no evidence to support the co-assembly of α5 with either α3 or α4 subunits (to form α3β2α5 or α4β2α5 receptors respectively). Consistent with previous publications (Brown *et al*., 2007; Baddick and Marks, 2011), deleting α5 had no effect on either the total number of receptors or the expression of any other subunit.

### [^3^H]-ACh release in the Hb and IPN

Both rats and mice had robust nicotine-induced [^3^H]-ACh release in the IPN, but not in the Hb. Consistent with previous findings in IPN synaptosomes (Grady *et al*., 2001,2009), deleting the β4 subunit – but not α5 or β2 – eliminated nicotine-induced ACh release. Grady *et al*. (2009) also measured the release of [^3^H]-ACh from IPN synaptosomes isolated from α2-, α4-, α6-, α7- and β3-KO mice and found that ACh-induced release was reduced in β3-KO mice but not in the other KO mice. Because cytisine – a partial agonist of β2-containing receptors and a full agonist of β4-containing receptors (Papke and Heinemann, 1994) – was as potent as nicotine in inducing [3H]-ACh release in the rat IPN, we conclude that – as in mice – β4-containing receptors mediate this release. The release of [3H]-ACh was calcium-dependent but TTX-insensitive, indicating that the primary source of calcium entry is presynaptic nACh receptors (Kulak *et al*., 2001), not voltage-gated calcium channels (Wonnacott, 1997; Kulak *et al*., 2001). Surprisingly, (and in contrast with the cortex and hippocampus), field stimulation with our standard pulse protocol induced only a marginal release of [^3^H]-ACh from the IPN in both rats and mice. However, IPN synaptosomes also require a higher concentration of [KCl]_o_ for inducing the release of ACh (40 mM; Grady *et al*., 2001) than cortical synaptosomes for the release GABA or striatal synaptosomes for the release of dopamine (20 mM; Grady *et al*., 2012), suggesting differences in the threshold for activating transmitter release. Alternatively, electrical stimuli may activate additional transmitter systems that exert inhibitory effects on the release of ACh. Indeed, the IPN contains a large population of GABAergic neurons (Kawaja *et al*., 1989; Lena *et al*., 1993; Hsu *et al*., 2013) and receives noradrenergic input from the LC, 5-hydroxytryptaminergic input from the raphe nuclei and dopaminergic input from the ventral tegmentum (Klemm, 2004; Lecourtier and Kelly, 2007; Bianco and Wilson, 2009; Kobayashi *et al*., 2013). However, neither the GABA_A_ receptor antagonist bicuculline nor the α_2_ adrenoceptor antagonist yohimbine affected electrically induced [^3^H]-ACh release.

Our data support and extend previous reports that despite its rich cholinergic innervation and the presence of postsynaptic nACh receptors, evidence of fast cholinergic synaptic transmission in the IPN remains controversial (Brown *et al*., 1983; Ren *et al*., 2011). Thus, selectively stimulating cholinergic axon terminals with optogenetics elicits fast responses mediated by synaptic ionotropic glutamate receptors; in contrast, continuous 5 s or tetanic light pulses (≥20 Hz for 20 s) elicit slow cholinergic effects mediated by nicotinic receptors (Ren *et al*., 2011). However, in slice preparations, MHb cells generated tonic trains of action potentials that averaged 5 Hz (Kim and Chang, 2005). Although applying ACh or glutamate caused rapid, short-lasting excitation of MHb neurons (McCormick and Prince, 1987), whether these neurons fire *in vivo* for sufficient duration at ≥20 Hz in order to cause the slow cholinergic effects observed by Ren *et al*. (2011) remains unknown.

### [^3^H]-NA release in Hb and IPN

[^3^H]-NA release differed from [^3^H]-ACh release in both rats and mice. First, nicotine-induced [^3^H]-NA release in both the IPN and Hb was TTX-sensitive, suggesting an effect mediated by preterminal – rather than presynaptic – nACh receptors (Wonnacott, 1997). In striking contrast, including TTX in the superfusion buffer had no effect on the nicotine-induced release of [^3^H]-ACh in the IPN. Secondly, electrically induced [^3^H]-NA release greatly exceeded nicotine-induced [^3^H]-NA release in both the IPN and Hb (and even more so in the hippocampus); in contrast, in the IPN, nicotine induced much more [^3^H]-ACh release than electrical stimuli.

The nicotine-induced [^3^H]-NA release in both the IPN and Hb required the presence of β2- and β4-containing receptors, as shown by the significantly reduced release in the respective KO mouse lines. In contrast, deleting the α5 subunit did not affect efficacy, but rendered nACh receptors less sensitive to nicotine-induced [^3^H]-NA release.

### Role of the α5 nACh receptor subunit

The α5 subunit is considered an ‘accessory’ subunit, as it requires both a β subunit and another α subunit (e.g. α4β2α5 or α3β4α5 receptors) to form a functional receptor (Ramirez-Latorre *et al*., 1996; Wang *et al*., 1996; Gerzanich *et al*., 1998). Consistent with our results, deleting the α5 subunit renders mice less susceptible to both nicotine-induced seizures (Salas *et al*., 2003) and the analgesic effects of nicotine (Jackson *et al*., 2010).

Similarly, nicotine – and to an even greater extent, ACh – activated recombinant human α3β2α5 nACh receptors expressed in *Xenopus laevis* oocytes more potently than α3β2 receptors (Gerzanich *et al*., 1998). On the other hand, chick α4β2α5 nACh receptors expressed in *Xenopus laevis* oocytes were less sensitive to nicotine than α4β2 receptors (Ramirez-Latorre *et al*., 1996; Fucile *et al*., 1997). Conversely, including α5 had little effect on the sensitivity of native and recombinant α3β4 (Fucile *et al*., 1997; Gerzanich *et al*., 1998; Fischer *et al*., 2005) and on native α4β2 nACh receptors (although the α-CtxMII–resistant component of dopamine release was largely inhibited in α5 KO mice; Salminen *et al*., 2004). Human (α4β2)_2_α5 receptors (i.e. receptors comprised of two α4 subunits, two β2 subunits and one α5 subunit) stably expressed in HEK cells were more potently activated by nicotine than (α4β2)_2_α4 receptors, but less potently than (α4β2)_2_β2 receptors (Kuryatov *et al*., 2008).

In mouse hippocampal synaptosomes, two types of nACh receptors have been proposed to mediate NA release: α6(α4)β2β3β4 and α6(α4)β2β3 receptors (Azam and McIntosh, 2006). Consistent with this report, we found that deleting α5 did not change the potency of nicotine at triggering [^3^H]-NA release in the mouse hippocampus, suggesting that the nACh receptors that modulate NA release system differ between the hippocampus and the Hb-IPN system. A similar conclusion has recently been reached by comparing the NA release in the rat hippocampus and frontal cortex (Kennett *et al*., 2012). The general consensus in the field is that the prefrontal cortex (Berridge and Waterhouse, 2003; Robertson *et al*., 2013), the hippocampus (see Oleskevich *et al*., 1989; Robertson *et al*., 2013) and the IPN (reviewed in Klemm, 2004; Lecourtier and Kelly, 2007; Bianco and Wilson, 2009) all receive noradrenergic input from the LC. Hence, either target-dependent effects on receptor expression, or projections that arise from diverse populations of brainstem noradrenergic neurons may account for the different types of receptors. The latter concept has recently been put forward by showing that noradrenergic neurons in the brainstem are comprised of four genetically distinct subpopulations (Robertson *et al*., 2013). Based on this new classification, future experiments may assign distinct populations of brainstem NA neurons with different properties to various brain regions, including the Hb-IPN system.

α5-KO mice do not exhibit an aversion to high doses of nicotine, and their nACh receptor-mediated signalling in the IPN (assessed by the number of Fos-positive cells) is reduced, suggesting a gain-of-function effect if α5 assembles into α3β4 receptors (Fowler *et al*., 2011). Consistent with this hypothesis, transgenic mice with targeted overexpression of *CHRNB4* have a strong aversion to nicotine and viral-mediated expression of the α5 D398N variant in the MHb – which reduces the function of α3β4 receptors – reversed this aversion to nicotine (Frahm *et al*., 2011). However, not only the α5 D398N variant but also WT α5 decreased the current amplitudes if added to α3β4 receptors expressed in *Xenopus laevis* oocytes (Frahm *et al*., 2011), and overexpressing the *CHRNA5*/*CHRNA3*/*CHRNB4* gene cluster created a mouse that was prone to nicotine consumption (Gallego *et al*., 2012). To date, no unifying mechanism has been proposed to explain these partially contradicting observations. Here, we found that (i) electrical stimuli – unlike nicotine – induce only a weak release of [^3^H]-ACh in the IPN, (ii) nicotine-induced [^3^H]-ACh release depends on β4- but not α5-containing nACh receptors, and (iii) deleting the α5 subunit reduces the potency of nicotine at inducing NA release in the mouse Hb-IPN. Together, these findings increase the probable complexity of the underlying mechanisms.

## Abbreviations

18-MC: 18-methoxycoronaridine
Hb: habenula
IP: immunoprecipitation
IPN: interpeduncular nucleus
KO: knockout
LC: locus coeruleus
MCA: mecamylamine
MHb: medial habenula
nACh receptor: nicotinic acetylcholine receptor
TTX: tetrodotoxin
WT: wild-type
